# Reduced egg shedding in nematode-resistant ewes and projected epidemiological benefits under climate change

**DOI:** 10.1016/j.ijpara.2019.06.008

**Published:** 2019-11

**Authors:** H. Rose Vineer, P. Baber, T. White, E.R. Morgan

**Affiliations:** a Veterinary Parasitology and Ecology Group, Bristol Veterinary School, University of Bristol, BS8 1TQ, UK; b Sheep Improved Genetics Ltd., Jersey Farm, Devonshire Gate, Tiverton EX16 7EJ, UK

**Keywords:** Faecal egg count (FEC), Resistance, Gastrointestinal nematodes, Peri-parturient rise (PPR), Estimated breeding values (EBVs), Climate change, Breeding, Parasite

## Abstract

Global livestock production is facing serious new challenges, including climate-driven changes in parasite epidemiology, and anthelmintic resistance, driving a need for non-chemotherapeutic methods of parasite control. Selecting for genetic resistance to gastrointestinal nematode infection could reduce reliance on chemical intervention and mitigate increases in parasite challenge due to climate change. Ewes of the composite Exlana breed with a range of estimated breeding values (EBVs) based on nematode faecal egg counts (FECs) were monitored during the peri-parturient period on two farms in southwestern England. Ewes with low EBVs (“resistant”) had lower FECs during the peri-parturient period than those with high EBVs (“susceptible”): the mean FEC was reduced by 23% and 34% on Farms 1 and 2, respectively, while the peak FEC was reduced by 30% and 37%, respectively. Neither EBV nor FEC were correlated with key performance indicators (estimated milk yield, measured indirectly using 8 week lamb weight, and ewe weight loss during lactation). Simulations predict that the reduced FECs of resistant ewes would result in a comparable reduction in infection pressure (arising from eggs shed by ewes) for their lambs. Furthermore, although the reduced FECs observed were modest, simulations predicted that selecting for nematode resistance in ewes could largely offset predicted future climate-driven increases in pasture infectivity arising from eggs contributed by these ewes. Selective breeding of the maternal line for nematode resistance therefore has potential epidemiological benefits by reducing pasture infectivity early in the grazing season and alleviating the need for anthelmintic treatment of ewes during the peri-parturient period, thus reducing selection pressure for anthelmintic resistance. These benefits are magnified under predicted future climate change. The maternal line warrants more attention in selective breeding programmes for nematode resistance.

## Highlights

• Exlana breed ewes were monitored for gastrointestinal nematodes during the peri-parturient period.
• Ewes selected for resistance when lambs produced fewer eggs as adults.
• There was no observed reproductive cost to resistance.
• Simulations predict that lambs of resistant ewes are exposed to reduced infection pressure.
• Nematode resistance in the female line could help mitigate the impact of climate change on infection pressure.

## Graphical abstract

**Figure:**
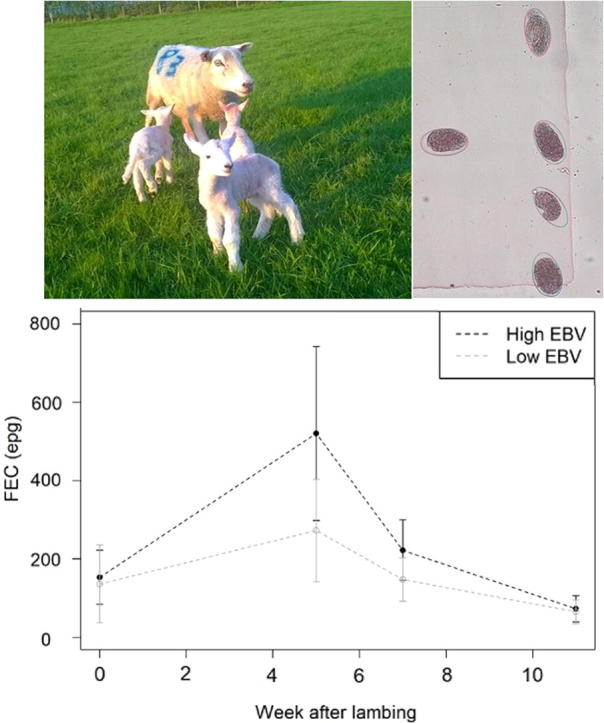

## 1 Introduction

Livestock producers have faced new challenges in parasitic disease control in recent decades due to climate-driven changes in the epidemiology of parasites, including the gastrointestinal nematodes that cause ovine parasitic gastroenteritis (van Dijk et al., 2008, Gethings et al., 2015). Furthermore, anthelmintic resistance is widespread (Kaplan and Vidyashankar, 2012, Rose et al., 2015a) and threatens the resilience and sustainability of small ruminant production enterprises (Coles et al., 2006). As a result, sheep producers worldwide are looking to alternative methods of nematode control (Charlier et al., 2018) such as bioactive forages (e.g. Lüscher et al., 2014), vaccination (e.g. Nisbet et al., 2016) and breeding for resistance to endemic nematode species (e.g. Bishop and Stear, 1997).

The observed impact of selecting individuals with low faecal egg counts (FECs) on gastrointestinal nematode epidemiology, for example to reduce infection pressure in subsequent years (Gruner et al., 2002), the relatively high heritability of FECs (Bishop et al., 1996, Stear et al., 1997, Bishop and Stear, 2001), and the genetic correlation between low FECs and productivity traits such as growth rate (Bishop et al., 1996, Stear et al., 1997) are encouraging. However, breeding for nematode resistance has so far focused largely on the male line, since selection of rams with low FECs allows genetic improvements to be passed on to a large number of offspring.

Ewes are known to increase nematode egg shedding in faeces during the peri-parturient period, around the time of lambing. This peri-parturient rise (PPR) in FECs provides an important source of eggs to contaminate pastures and increase subsequent infection pressure for lambs. Many farms treat ewes around the time of lambing to minimise contamination of lambing paddocks. Due to the contribution of the eggs produced during the PPR to the nematode population infecting lambs each grazing season, however, there are concerns that this strategy may select heavily for drug-resistant nematodes (Leathwick et al., 1995). Furthermore, sub-therapeutic doses of anthelmintics may be transferred to the lamb via the ewe’s milk, further selecting for resistance (Leathwick et al., 2015). Selective breeding of ewes for attenuated PPR could reduce the need to treat ewes and lambs, and play an important role in slowing the development of anthelmintic resistance. These benefits are unlikely to be realised by focusing uniquely on terminal sire breeds in cross-bred meat lamb production systems, since it is the maternal line that generates early season pasture contamination.

Estimated Breeding Values (EBVs) are used by commercial breeders to select for desirable traits as they take into account the effects of genotype and environment on the observed phenotype (Mrode and Thompson, 2005). Hence, this approach offers a practical method for genetic improvement within breeds where the breeding population available for selection is maintained across multiple management groups and farms (i.e. environments). For gastrointestinal nematode infection, EBVs are evaluated using FECs from lambs >18 weeks of age, although an IgA saliva test is under development (Shaw et al., 2012). The value of EBV assigned to ewe lambs is unknown in terms of reducing future PPR. In addition, it is unclear whether enhanced immunity to nematodes following lambing could carry costs in terms of milk output or ewe weight loss. This is a risk since the energy and protein costs of immunity may trade off against those of growth and reproduction (Kidane et al., 2010, Rauw, 2012). Selecting for resistance to nematodes might also trade off against an ability to resist other parasites and pathogens (Ezenwa et al., 2010). Improved understanding of the relationship between EBV for nematode FEC and the PPR could help to define targets for selective breeding, and quantify the expected epidemiological benefits of selective breeding strategies through reduced early season pasture contamination.

A potential limitation to the strategy of breed improvement for parasite resistance is that it takes many years to attain goals, yet is necessarily tied to performance under current environmental and management conditions. Where the epidemiological situation is changing, as for gastrointestinal nematodes under climate warming (van Dijk et al., 2008, Morgan and Wall, 2009), current breeding efforts might not be well matched to future conditions. Epidemiological models developed for gastrointestinal nematodes in sheep predict changes in both the magnitude and seasonal patterns of challenge as a function of temperature and rainfall (Rose et al., 2015b, Rose et al., 2016). Predictive models could be used to determine likely future changes in the epidemiological context of selective breeding programmes, to help inform selection goals.

In this paper we aim to determine whether selection of ewe lambs for reduced FEC leads, in later life, to lower egg output during the PPR. Further, we explore the benefit of reduced PPR in terms of lower gastrointestinal nematode infection pressure for lambs under current and future climate scenarios.

## 2 Materials and methods

### 2.1 Field data collection

Exlana ewes on two separate farms in southwestern England (referred to as Farm 1 and Farm 2) were monitored in the spring of 2014 as part of an ongoing commercial breed improvement programme (Sheep Improved Genetics Ltd – http://www.sig.uk.com/). The Exlana is a composite hair breed sheep based on more than 14 breeds selected for their favourable or low input features, such as the Wiltshire Horn, Barbados Blackbelly and Lleyn. The dominant gastrointestinal nematode species in this region is *Teladorsagia circumcincta*. *Haemonchus contortus* infection was not observed or suspected on these farms.

Ewes were selected for inclusion across a wide range of EBVs for FECs, with similar numbers of high (positive) and low (negative) scores. EBVs were estimated by Signet (http://www.signetfbc.co.uk/) using the Best Linear Unbiased Prediction (BLUP) method on FECs taken when the ewes were 5–6 months old. BLUP takes into account individual FECs, environmental effects such as management group, and pedigree (Mrode and Thompson, 2005). EBVs for nematode FEC are inversely related to resistance to gastrointestinal nematode infection: low (negative) EBVs indicate higher levels of resistance, and high (positive) EBVs indicate lower levels of resistance. A total of 47 ewes were monitored on Farm 1, and 65 ewes on Farm 2.

All ewes lambed over a 10-day period in mid-March, and within each farm were managed together for the duration of the study. On Farm 2, monitored ewes were all of similar age (2 or 3 years), and gave birth to twins (with the exception of one triplet-bearing ewe). This restriction was intended to reduce variation in FEC due to, inter alia, unequal resource allocation trade-offs among ewes feeding different numbers of lambs, which could modify the expression of immunity and hence FEC during the PPR (Coop and Kyriazakis, 1999). Equivalent restriction was not possible on Farm 1 due to limited numbers of ewes. On both farms, ewes and lambs grazed ad libitum on pasture composed primarily of *Lolium perenne* (perennial ryegrass)*.* No supplementary feed was given.

Around the time of lambing, and on three further occasions in the next eight (Farm 1) and 11 (Farm 2) weeks, individual faecal samples were taken from ewes per rectum by the farmer. Not all ewes provided a sample on each occasion. On Farm 2, lambs were weighed at approximately 8 weeks of age as a measure of dam milk production, and ewes were weighed in late pregnancy and at the end of the study. Faecal samples were sent to the laboratory by post where they were stored at 8 °C, and FECs were conducted within 5 days using a modified McMaster method with a detection limit of 10 eggs per gram of faeces (epg). Trichostrongylid nematode eggs, *Nematodirus* spp. eggs and coccidia oocysts were counted separately. Raw counts were used for statistical analysis and transformed to epg for data presentation. For each farm, the week of peak FEC was extracted for individual ewes with at least three FECs over the four sampling periods to estimate the timing and magnitude of the peak FEC during the PPR. For all other analyses, the complete dataset was used, except 11 ewes that were excluded from the dataset for Farm 2 as FECs were only available for the first sampling period. A further four ewes with a single FEC in either week 5 or 7 were also identified on Farm 2. However, these ewes were not excluded as these FECs fell within the plausible peak of the PPR.

### 2.2 Impact of phenotypic selection for resistance on the observed PPR

The relationship between the FEC-EBV estimated at 5–6 months of age and observed FEC in individual ewes around lambing was assessed by a Spearman rank correlation, using the peak FEC per ewe, and average FEC per ewe over all sampling occasions. One-way Kolmogorov-Smirnov tests were applied to these mean and peak FECs for ewes in low and high EBV groups to determine whether these observed FECs were drawn from the same distribution.

Differences between the average FEC at each sampling point, and over the entire duration of the study, were compared between high (positive or zero) and low (negative) EBV groups using bootstrapping, following Morgan et al. (2005). Thus, individual FECs in each group were resampled with replacement, and sample size of the most numerous group truncated to match that of the smaller group, to compensate for overdispersion in FECs. On Farm 1, 18 ewes were in the high (positive or zero) EBV group and 29 in the low (negative) EBV group, and corresponding numbers on Farm 2 were 31 and 23. Monte Carlo simulation was used to generate 10,000 replicates, where individual bootstrapped counts in the low EBV group were subtracted from those in the high EBV group, and the mean difference calculated. The proportion of simulations where low EBV ewe counts were lower than high EBV ewe counts was then calculated from the 10,000 replicate simulations. A statistically significant difference in mean FEC was considered to be present where the difference between high and low EBV ewe counts was equal to or below zero in fewer than 5% of replicates, corresponding to the traditional *P* < 0.05 cut-off value.

### 2.3 Association between EBV and key performance indicators

To evaluate whether enhanced immunity against nematodes, manifested as reduced FECs, came at the cost of reduced resources for milk production or for maintenance of body weight, a Spearman rank correlation was used to assess associations between EBVs, FECs, and indicators of performance, i.e. mean lamb weight per dam at 8 weeks of age, and ewe weight loss between late pregnancy and the end of the study.

### 2.4 Predicted impact of attenuated PPR on lamb infection pressure

**Fig. 1:**
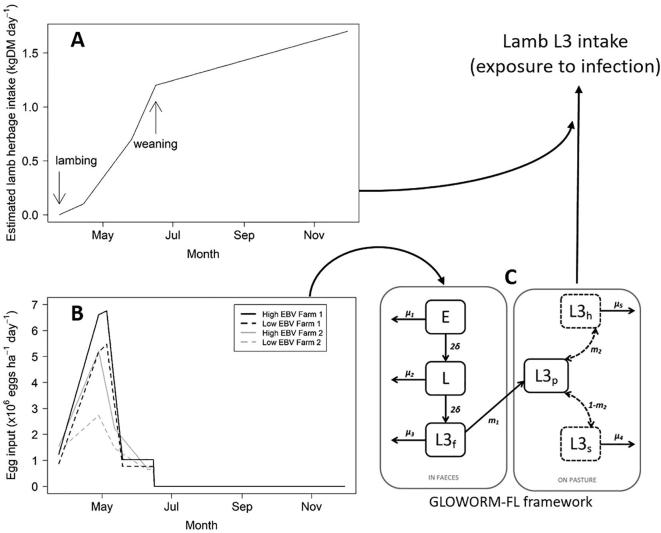
Conceptual diagram of model simulations estimating the exposure of lambs on two farms in southwestern England to *Teladorsagia circumcincta* or *Haemonchus contortus* infective larvae (L3) contributed by ewes during the peri-parturient period. The GLOWORM-FL model of the free-living stages of gastrointestinal nematodes (Rose et al., 2015b) requires daily faecal egg counts as input (e.g. the observed faecal egg counts of ewes during the peri-parturient period from the two farms in 2014, B) as well as daily temperature and rainfall data (not shown). The eggs (E) develop (δ) through the pre-infective first and second larval stages (L) to infective larvae in the faeces (L3f). The L3 then migrate out (m_1_) onto pasture (L3p) where they migrate vertically (m_2_) between the soil (L3s) and herbage (L3h). All free-living stages die at stage-specific rates (μ_i_). All parameters are dependent on temperature and/or rainfall as described by Rose et al. (2015b). The model provides the daily number of eggs, larvae in faeces, and infective larvae on pasture as output, with the number of infective larvae on pasture further subdivided to give the numbers on herbage and in soil. These outputs can be used directly or subjected to further analysis. For example, the number of L3 on herbage per hectare estimated by the model can then be multiplied by the daily lamb herbage intake (A) to estimate the daily L3 intake, which is a direct estimate of exposure of lambs to infection arising from eggs excreted by ewes. DM, dry matter.

The impact of the observed reduction in PPR on pasture contamination and lamb exposure to third-stage infective larvae (L3) arising from eggs deposited by ewes was simulated using the GLOWORM-FL model, which tracks the climate-dependent population dynamics of the free-living stages of *T. circumcincta* (Fig. 1C; Rose et al., 2015b)*.* The model was implemented in R v3.6.0 (R Core Development Team, 2019. R: A language and environment for statistical computing. R Foundation for Statistical Computing, Vienna, Austria. http://www.R-project.org/) using the *lsoda* function in the *deSolve* package (Soetaert et al., 2010).

Model input required daily temperature and rainfall data. Mean daily air temperature (°C) and total daily precipitation (mm) for the grazing season (24 March 2014 to 30 November 2014) were extracted from the EOBS gridded dataset v11.0 (Haylock et al., 2008) using the *ncdf4* (Pierce, D., 2013. ncdf4: Interface to Unidata netCDF (version 4 or earlier) format data files. R package version 1.9. http://dwpierce.com/software) and *chron* (James, D., Hornik, K., 2014. chron: Chronological objects which can handle dates and times. R package version 2.3–45) packages in R. Climatic time series were continuous except for one missing value in the precipitation dataset, which was replaced with 0 mm to match rainfall values on adjacent days.

Model input also required the number of eggs deposited on pasture per hectare, per day, between lambing and weaning, which was estimated as the daily mean group FEC for the high and low EBV ewes, respectively, multiplied by a representative stocking rate of five ewes per hectare (ha) and 2000 g of faeces produced daily (Kao et al., 2000; Fig. 1B). Linear interpolation was performed on the raw FEC data using the *approxfun* function in R, to estimate daily group FEC values. Ewe FEC input after weaning was reduced to 0 to simulate removal of ewes from the pasture. Model simulations track the fate of these eggs excreted by ewes during the PPR in FECs (i.e. egg development and mortality, and larvae development, mortality and migration onto herbage) and provide daily estimates of the number of infective larvae on herbage (L3h) per unit area (per hectare in the present study).

From the model output, hypothetical exposure of lambs to infection by L3h contributed by high EBV and low EBV ewes on both farms were estimated for the period between birth to weaning, and between birth to slaughter at the end of the grazing season (assuming lambs remained on the lambing paddocks for the duration). For this, L3h kg DM^−1^(dry matter) ha^−1^ was estimated using the L3h ha^−1^ (*L3h*) from the model output and a representative herbage biomass (*B*) of 2000 kg DM ha^−1^ (Kao et al., 2000). This was then multiplied by the predicted herbage dry matter intake (DMI), which was assumed to increase from 0 kg DM day^−1^ at birth to 1.2 kg DM day^−1^ at weaning (Hynes 2013), and thereafter increased linearly from 1.2 kg DM day^−1^ to 1.7 kg DM day^−1^ (EBLEX, 2014; Fig. 1A).

$$L3i=\frac{L3h}{B}DMI$$

Eggs deposited by lambs were not included in the simulations, since the aim was to track the consequence of eggs produced from ewes on lamb infection pressure.

### 2.5 Predicted potential of a reduced PPR to mitigate climate change impacts

Gastrointestinal nematode infection pressure is predicted to increase under climate change (Rose et al., 2015b, Rose et al., 2016). Additional model simulations were therefore run to explore the potential for the reduced PPR in FECs in resistant ewes to mitigate increased infection pressure under climate change, under the assumption that the nematodes would not adapt their thermal niche to the changing climate within this timeframe. Simulating the reduced PPR in resistant ewes considers lambs’ exposure to infection originating from ewes during the peri-parturient period, such as in management systems where ewes are removed from the lambs at weaning but lambs remain on the contaminated pasture.

Eight factor combinations were simulated in a 2^3^ factorial design considering the nematode species (*T. circumcincta* and *H. contortus*), host resistance status (susceptible and resistant), and climate (historic and future high emissions scenario). Although *T. circumcincta* is currently more common in the study area than *H. contortus*, simulations suggest increasing *H. contortus* in northern Europe in future (Rose at al., 2016), and breeding strategies should be future-proofed against that risk.

Simulations assumed ewes grazed a single pasture where they lambed from day 56 (end of February), and were removed at weaning on day 181 (end of June) when lambs were approximately 12–14 weeks of age.

Representative FEC profiles for *T. circumcincta* were defined using the high and low EBV ewe FECs observed in this study on Farm 2. *Haemonchus contortus* FECs are typically several-fold higher than *T. circumcincta,* therefore the same FEC profiles used for *T. circumcincta* were adapted for *H. contortus* using a multiplication factor of 5. Linear interpolation was performed on the raw FEC data using the *approxfun* function in R, to estimate daily group FEC values.

Historic climate data for a baseline period (1970–1998) and future projected data for 2071–2099 under the RCP8.5 scenario were obtained for southwestern England from the Coupled Model Intercomparison Project Phase 5 (CMIP5; Taylor et al., 2012) as described by Rose et al. (2015b). In order to calculate mean seasonal pasture contamination, there was no carry-over of infective larvae on pasture between years, and model output is presented as a mean of the 29  years simulated.

The impact of resistance, climate change, and resistance plus climate change was estimated by calculating the percentage change in annual pasture contamination compared with baseline simulations of susceptible (high EBV) ewes using the historic climatic data.

## 3 Results

### 3.1 Field data collection

**Fig. 2:**
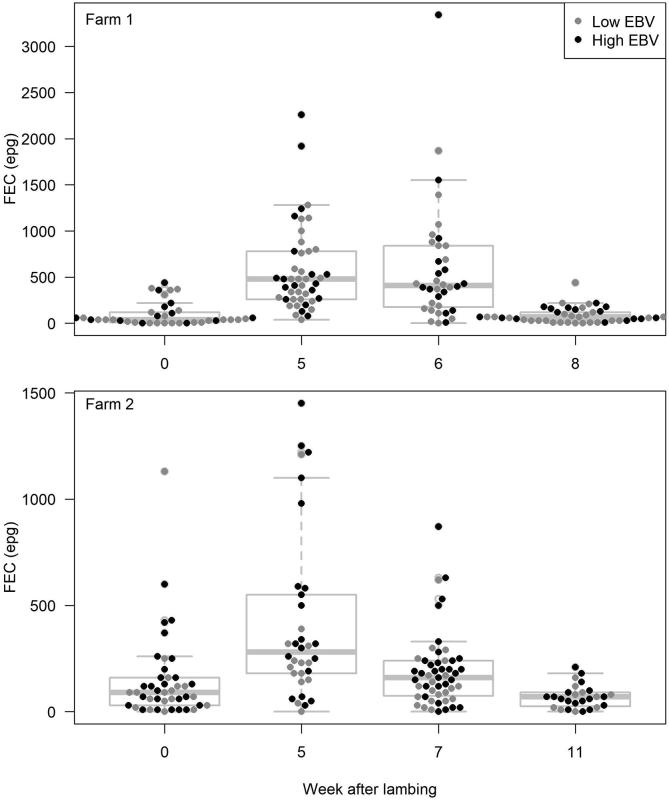
The distribution of individual ewe faecal egg counts at each sampling point for Farm 1 and Farm 2 (both in southwestern England). Boxes show the interquartile range with an intersecting median line. Whiskers display 1.5× the interquartile range. Individual ewe faecal egg counts are shown as overlaid black (high estimated breeding value group) and grey (low estimated breeding value group) points. epg, eggs per gram of faeces.

An average of 3.5 faecal samples per ewe on Farm 1 (164 FECs on 47 ewes), and 2.6 on Farm 2 (166 FECs on 65 ewes) were analysed during the peri-parturient period. Individual ewe FEC and EBV data for both farms are provided in Supplementary Tables S1 and S2. On both farms, the average FEC increased after lambing, peaking at subsequent sampling occasions, before declining (Fig. 2). FECs peaked approximately 5 weeks after lambing (Farm 1, week 0 = 2, week 5 = 24, week 6 = 16, and week 8 = 0 ewes; Farm 2, week 0 = 9, week 5 = 21, week 7 = 8 and week 11 = 0 ewes; Supplementary Fig. S1). The median maximum individual FEC was 510 epg on Farm 1 (range 50–3340) and 280 epg on Farm 2 (range 20–1220; Fig. 2).

The coccidian oocyst count also peaked on Farm 2 in week 5 at 521 oocysts per gram of faeces (range 0 to 2640), and tended to be higher in ewes also shedding high numbers of nematode eggs (*r*_s_ = 0.31, *P* = 0.058). On Farm 1, peak coccidian oocyst counts were lower than on Farm 2 (215 per gram, range 0 to 1200), and there was no correlation between them and nematode egg counts.

### 3.2 Impact of phenotypic selection for resistance on the observed PPR

**Fig. 3:**
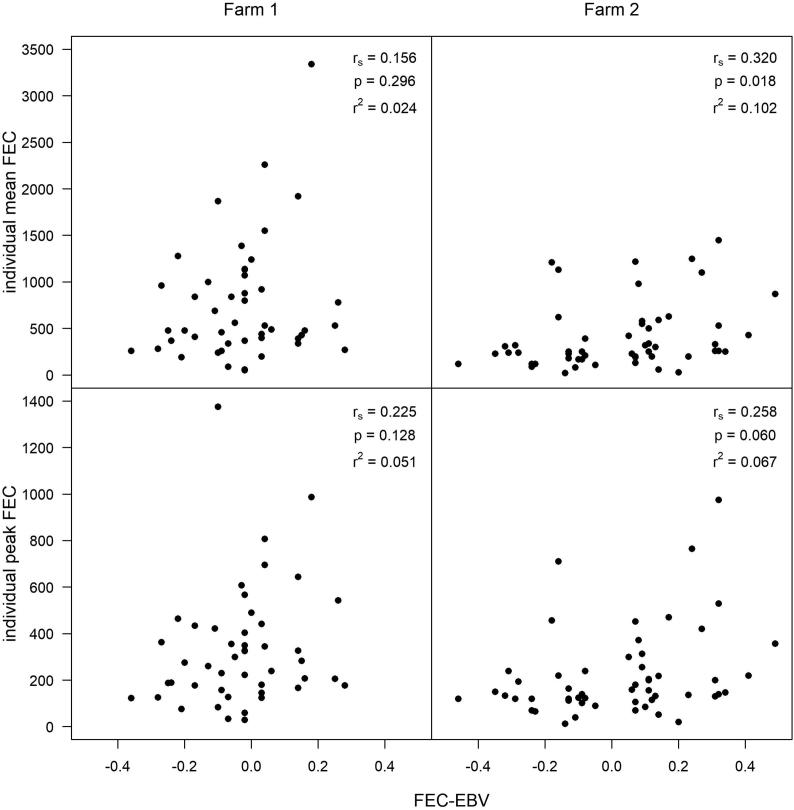
Individual ewe mean and peak faecal egg counts correlated with estimated breeding values, for Farm 1 and Farm 2 (both in southwestern England).

There was a weak, positive relationship between mean FEC and EBV (*r*_s_ = 0.258, *P* = 0.060, *r*^2^ = 0.067) and peak FEC and EBV (*r*_s_ = 0.320, *P* = 0.018, *r*^2^ = 0.102) on Farm 2, where EBVs ranged from 0.46 to 0.49 (Fig. 3, Supplementary Fig. S2). There was greater uncertainty on Farm 1 (max FEC: *r*_s_ = 0.156, *P* = 0.296, *r*^2^ = 0.024, and mean FEC: *r*_s_ = 0.225, *P* = 0.128, *r*^2^ = 0.051), where the range of EBVs was smaller (−0.36 to 0.28; Fig. 3, Supplementary Fig. S2), and r^2^ was low for all correlations (Fig. 3). The distribution of FECs observed in low and high EBV ewes overlapped (Fig. 2). There was a significant difference in the distribution of mean FECs (D = 0.339, *P* = 0.048) and peak FECs (D = 0.384, *P* = 0.020) between the low and high EBV ewes on Farm 2, but not on Farm 1 (D = 0.251, *P* = 0.247, and D = 0.213, *P* = 0.366, respectively).

**Fig. 4:**
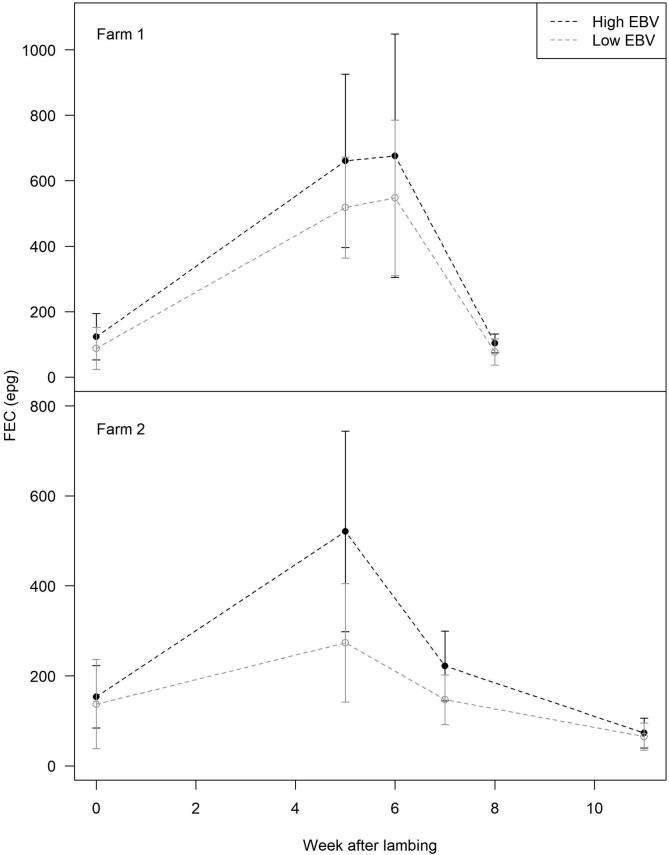
Bootsrapped mean faecal egg counts (points) and 95% confidence intervals (error bars) for high estimated breeding value; black) and low estimated breeding value (grey) ewes on Farm 1 and 2 (both in southwestern England).

**Fig. 5:**
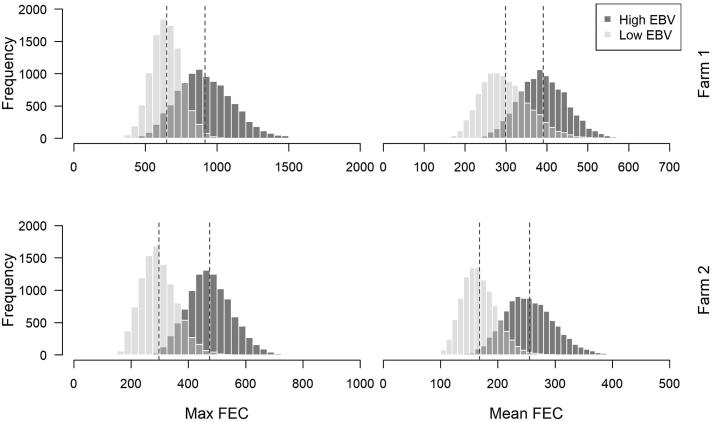
Bootstrapped mean maximum faecal egg counts and mean faecal egg counts over the peri-parturient rise in faecal egg counts) for Farm 1 and Farm 2 (both in southwestern England). Counts for low estimated breeding value ewes are shown in light grey, and high estimated breeding value ewes are shown in dark grey. Vertical ablines indicate the mean values for the 10,000 bootstrapped samples in each dataset.

Bootstrapped mean FECs over the duration of the PPR were, on average, 23% lower on Farm 1 and 34% lower on Farm 2, in low EBV ewes than high EBV ewes (Table 1; Fig. 4, Fig. 5). The difference in FECs between low EBV and high EBV ewes was more pronounced around the peak of the PPR (Fig. 4). Significant differences were observed in week 5 (*P* = 0.036) and week 7 (*P* = 0.061; tending towards statistical significance) on Farm 2, although not on Farm 1. Bootstrapped peak FECs were 30% and 37% lower in low EBV ewes compared with high EBV ewes on Farms 1 and 2, respectively (Table 1; Fig. 4, Fig. 5). Overall, on Farm 2, low EBV ewes had lower mean and peak FECs than high EBV ewes in 95–97% of Monte Carlo simulations. The effect size was less pronounced on Farm 1 (87–89%; Table 1).

**Table 1:** Monte Carlo simulation estimates of the mean maximum faecal egg count (faecal egg counts (eggs per gram of faeces) and mean faecal egg counts over the course of the peri-parturient rise (PPR; in faecal egg counts, epg) for high (nematode susceptible) and low (nematode resistant) estimated breeding value ewes on two farms in southwestern England, the proportionate reduction in mean faecal egg counts and max faecal egg counts in low EBV ewes compared with high estimated breeding value ewes, and the proportion of simulations where the simulated means for low estimated breeding value ewes were lower than for high estimated breeding value ewes.

		Low EBV	High EBV	Proportion reduction in FEC (1-Low EBV/High EBV)	Proportion simulations where lowEBV ≤ highEBV
Farm 1	Peak FEC (S.D.)	646 (438)	918 (796)	0.296	0.894 (*P* = 0.106)
	Mean FEC (S.D.)	300 (236)	391 (245)	0.233	0.866 (*P* = 0.134)

Farm 2	Peak FEC (S.D.)	297 (283)	472 (368)	0.371	0.967 (*P* = 0.033)
	Mean FEC (S.D.)	168 (137)	255 (203)	0.341	0.954 (*P* = 0.046)

### 3.3 Association between EBV and key performance indicators

On Farm 2, for which ewe and lamb live-weight data were available (Supplementary Table S3), ewes lost an average of 6.27 kg (±S.D. 5.12 kg) between lambing and weaning (reducing from 56.8 2 kg (±S.D. 4.58 kg) to 50.55 kg (±S.D. 6.00 kg)). Ewe weight loss tended to be higher in ewes that produced higher maximum FECs, though there was substantial unexplained variability (*r*_s_ = 0.281, *P* = 0.044, *r*^2^ = 0.079), but was not correlated with EBV (*r*_s_ = −0.074, *P* = 0.603, *r*^r^ = −0.005) nor average FEC over the PPR (*r*_s_ = 0.235, *P* = 0.093, *r*^2^ = 0.055).

Lambs weighed an average of 21.26 kg (±S.D. 3.41 kg) at 8 weeks of age and ewes produced a total 8 week lamb weight of 35.13 kg (±S.D. 9.28 kg). Eight week lamb weight was not significantly correlated with maximum ewe FEC (*r*_s_ = −0.044, *P* = 0.756, *r*^2^ = −0.002), mean ewe FEC (*r*_s_ = −0.132, *P* = 0.352, *r*^2^ = −0.017), nor FEC EBV (*r*_s_ = −0.181, *P* = 0.197, *r*^2^ = −0.033).

### 3.4 Predicted impact of attenuated PPR on lamb infection pressure

Using the observed FECs for low and high EBV ewes on Farms 1 and 2 as input, together with historic climatic data and predicted lamb herbage intake, lamb exposure to *T. circumcincta* infection was simulated. The observed 23% reduction in FEC in low EBV groups compared with high EBV ewes on Farm 1 translated to a similar simulated reduction in lamb exposure of 22% in the period between lambing and weaning, and over the course of the grazing season. Similarly, the observed 34% reduction in ewe FECs on Farm 2 translated to a simulated reduction in lamb exposure of 41% in the period between lambing and weaning, and 39% over the course of the grazing season (Supplementary Figs. S3 and S4, Supplementary Table S4).

### 3.5 Predicted potential of a reduced PPR to mitigate climate change impacts

Using historic climatic data for southwestern England, the observed 34% reduction in faecal egg output from low EBV compared with high EBV ewes on Farm 2 resulted in a simulated reduction in predicted exposure of lambs to infective larvae (number of L3 ingested) of between 34% and 38% (Table 2).

**Table 2:** The change in predicted pasture contamination (numbers of infective larvae on pasture) of two farms in southwestern England contributed by resistant ewes (low estimated breeding value) under historic climatic conditions, and susceptible (high estimated breeding value) and resistant (low estimated breeding value) ewes under a future climate change scenario, compared with a baseline of susceptible ewes (high estimated breeding value) under historic climate conditions.

	Historic climatic conditions		2080s (RCP8.5 climate change scenario)	
Species	High EBV	Low EBV	High EBV	Low EBV
*Haemonchus contortus*	N/A (baseline)	−37.77%	+69.20%	+9.01%
*Teladorsagia circumcincta*	N/A (baseline)	−33.50%	+47.14%	+4.73%

Compared with the baseline simulations of susceptible (high EBV) ewes under historic climatic conditions, both *H. contortus* and *T. circumcincta* total pasture contamination increased under the future RCP8.5 scenario of climate change (Table 2).

**Fig. 6:**
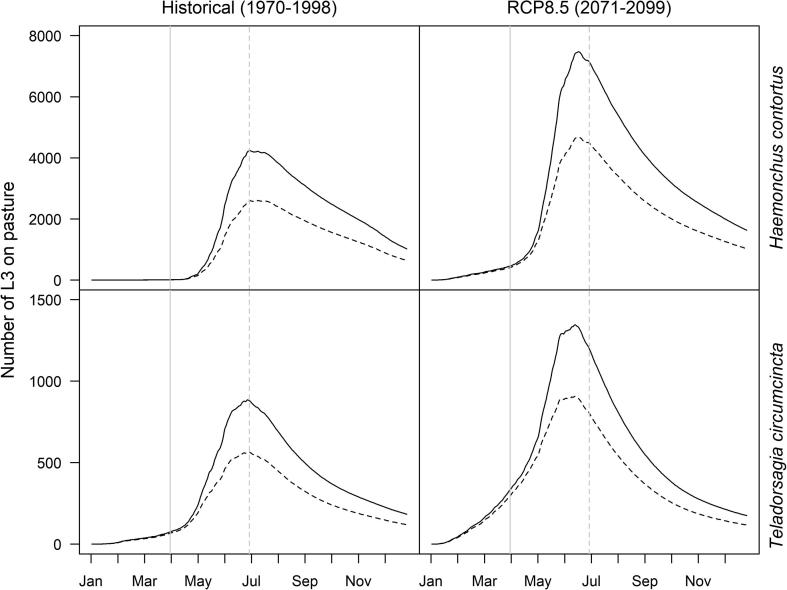
Simulated average numbers of third-stage infective larvae (L3) on pasture arising from eggs deposited by susceptible (solid line) and resistant (dashed line) ewes during the peri-parturient rise in faecal egg counts. Simulations were repeated for *Haemonchus contortus* and *Teladorsagia circumcincta* under historic and future climatic conditions.

Introducing resistant (low EBV) ewes was predicted to mitigate the impact of climate change, bringing pasture contamination back in line with historic baseline predictions for susceptible (high EBV) ewes (Table 2, Fig. 6).

## 4 Discussion

Ewes on two commercial farms were evaluated for resistance to gastrointestinal nematode infection as ewe lambs as part of a breed improvement programme, using EBVs based on FECs, and monitored after lambing to assess the impact of genetic status on nematode egg output during this critical period, when egg counts typically show a transient rise (the PPR). The impact of an attenuated PPR on the simulated exposure of lambs to infection arising from eggs excreted by ewes during the peri-parturient period, and the potential for resistant ewes to reduce future impacts of climate change on infection pressure, was simulated.

Resistance to gastrointestinal nematodes is largely a result of an acquired IgA-mediated regulation of worm fecundity after 3 months of age whereas variation in worm burden is not thought to be under genetic control in older lambs (Stear et al., 1997, Stear et al., 1999). As lamb meat production is focused on maximising growth rates up to around 6 months of age, lambs bred from resistant ewes and rams will only benefit from any heritable resistance in their final few weeks of life. Bishop et al. (1996) observed strong maternal effects, which decreased significantly in magnitude between 1 and 6 months of age, on the FECs of Scottish Blackface lambs. The role of maternal effects and the resistance of the ewe are therefore important in reducing exposure to infection in the first few months of a lamb’s life (Williams et al., 2010).

Although the correlation between EBV and FEC was weak on Farm 2 and non-significant on Farm 1, discretizing the data into low (negative values) and high (positive values) EBV ewes revealed that a low EBV was associated with reduced egg output during the peri-parturient period. Peak egg output was reduced by 30–37% in low compared with high EBV ewes, and overall egg output during the peri-parturient period was reduced by 23–34%. EBVs evaluated at 5–6 months of age may therefore be an appropriate phenotypic parameter to select for resistance to nematodes in maternal lines.

The method of calculating EBVs based on FECs at 5–6 months of age was chosen by the participating farms as this supports current UK levy board breeding services recommendations for estimating FEC EBVs (Agriculture and Horticulture Development Board’s (AHDB) Signet Breeding Services: www.signetfbc.co.uk). Previous studies have used a range of methods to evaluate resistance, e.g. FECs taken at 12 months of age (Williams et al., 2010), and multiple FECs taken from lambs throughout the first grazing season (Bishop et al., 1996). However, evaluating individual ewes for resistance at 12 months of age or later may not be practical in many commercial sheep production systems where replacement ewes are chosen at a much earlier stage in their first grazing season, and multiple individual FECs are labour intensive and may not be perceived by farmers as economically viable, especially for larger flocks.

Simulations tracking the development, survival and migration of L3 from eggs deposited on pasture showed that the reduction in egg output of low EBV (resistant) ewes compared with high EBV (susceptible) ewes translated into a similar reduction in exposure of lambs to L3. The small variation observed is due to interactions between egg deposition and weather conditions, which determine the development success of eggs on pasture. The predicted reduction in pasture contamination on fields grazed by “resistant” ewes is supported by the observation of Williams et al. (2010) that numbers of *Trichostrongylus colubriformis* infective larvae on pasture grazed by susceptible (control) Romney ewes during the peri-parturient period were over four times higher than on pasture grazed by resistant Romney ewes. Subsequent infection of lambs amplifies nematode populations on pasture, and survival of infective larvae over winter carries infection over to the next grazing season, magnifying onward epidemiological impacts of selective breeding on egg output during the PPR (Bishop and Stear, 1997).

Based on a conservative mean establishment rate of 12.7% (to account for the impact of developing acquired immunity on establishment rates; Gaba et al., 2006) the observed decrease in egg output by ewes in the low EBV group compared with the high EBV group, and the simulated fate of these eggs throughout the grazing season, could result in 826 and 1778 fewer adult worms in lambs on Farms 1 and 2, respectively, in the period between lambing and weaning. Therefore, the observed reduction in FECs in this study, and simulated reduction in pasture contamination, could result in meaningful reductions in subsequent pasture contamination by lambs, and substantially lower nematode burdens by the end of the grazing season. For example, a 4 year study of set-stocked groups of ‘resistant’ and ‘susceptible’ rams demonstrated that pasture contamination was significantly reduced, as confirmed using tracer lambs, and that worm burden was reduced by 99% in the ‘resistant’ group compared with the ‘susceptible’ group (Gruner et al., 2002).

Mounting an immune response is energetically expensive (Rauw, 2012) and the potential for unfavourable trade-offs between resources allocated to resistance to parasites and pathogens and other desirable traits should be considered in any breeding programme. In the present study, the reduced FEC in low EBV ewes was not associated with any sacrifice in production in terms of lamb growth or ewe weight loss. However, the sample size was relatively small compared with other studies that have reported such an association. For example, using a dataset of over 1400 FECs from more than 400 ewes, Bishop and Stear (2001) observed a positive genetic correlation of 0.24 between Scottish Blackface ewe FECs and 4 week weights of lambs, which may indicate a trade-off between milk production and resistance to gastrointestinal nematodes.

In this study, the high and low EBV ewes and their lambs were grazed on the same paddocks on both farms and shared a common environment. Therefore, the overall exposure to infection was similar for all lambs and the lambs born to high EBV ewes benefitted from the reduced FEC of co-grazing low EBV ewes. Given the negative association between worm burden and daily live weight gain (Mavrot et al., 2015), selecting for low EBV ewes to improve resistance across the entire flock could theoretically result in 8 week weights greater than observed in this study, as exposure to infective larvae would be reduced, in line with the model predictions presented here.

Previous modelling studies have demonstrated the potential for carry-over of L3 pasture contamination from year to year, to magnify the benefits of breeding for resistance (Bishop and Stear, 1997). However, none have explored the potential for resistance to mitigate climate-driven increases in infection pressure. The simulations presented in this study, under the assumption of equal FEC input, no parasite adaptation to a changing climate (Rose Vineer et al., 2016), and the modest decrease in FECs observed in low EBV compared with high EBV ewes, predicted that pasture infectivity could be reduced to historic levels by introducing resistant ewes.

Breeding for resistance shows considerable potential to mitigate the impact of climate change and offers a sustainable alternative to chemical interventions. Furthermore, the predicted benefit of breeding for nematode resistance in the maternal line is relevant to other novel approaches to gastrointestinal nematode control that achieve similar reductions in FECs. For example, Nisbet et al. (2016) achieved a 45% decrease in egg output during the peri-parturient period in ewes vaccinated against *T. circumcincta*. Notably, the observed decrease in egg output achieved by vaccination is greater than the average decrease achieved in the present study through selective breeding. Despite the relatively smaller reduction in FECs in the present study, the predicted benefits of this reduction in the context of reduced pasture contamination and reduced lamb exposure to infection lend support to the future role of vaccination in controlling gastrointestinal nematodes. Selective breeding might also enhance vaccine effectiveness in the field, as indicated by breed differences in responsiveness to a *T. circumcincta* vaccine (Gonzalez et al., 2019).

The simulations presented here considered only the impact of climate on the free-living component of the nematode life cycle as this is now well-characterised (Rose et al., 2015b) while there remains considerable uncertainty in the host’s response to infection (Saccareau et al., 2017), and in the drivers of hypobiosis (e.g. Capitini et al., 1990, Troell et al., 2006). Future insights into these processes could be incorporated into the model used here to assess their likely impact on predictions under current and future climates.

Pasture infectivity is highly variable from year to year due to the impact of variable weather conditions on the survival and development of the free-living stages (Rose et al., 2015b). It is therefore recommended that field trials evaluating novel approaches to gastrointestinal nematode control are designed with this uncertainty in mind and that longitudinal, multi-year trials are attempted where possible. The model presented here can help to design efficient field trials, by exploring different scenarios in more detail and selecting key areas for empirical confirmation.

In this study, similar qualitative patterns were observed on both farms, although differences in FECs between high and low EBV ewes at times of peak egg output on Farm 1 were more elusive. There was a statistically significant difference between the distributions of low and high EBV ewe mean FECs and maximum FECs on Farm 2, but not Farm 1. Similarly, the *P* values estimated by Monte Carlo simulation tended to be higher than the traditional acceptance threshold of 0.05 on Farm 1. This is reflected in the overlapping distributions of FECs in high and low EBV ewes. This is, to an extent, expected given the tendency for negative binomial parasite distributions within host populations. However, this could also be due to the lower sample size, narrower range of EBVs, and especially the looser inclusion criteria for the observed group on Farm 1, with ewes not managed in groups of similar age or number of lambs born, due to limited availability of ewes. Higher variability between ewes in factors affecting FECs during the PPR, such as individual fitness, nutrition or infection rate, or the resource demands of variable numbers of lambs, is likely to decrease the power of statistical comparisons between groups, and confound results. It is recommended that future studies, as far as possible, match ewes on potentially important criteria such as number of lambs, in order to increase study power. Effects of genetic resistance to nematode infection act through enhanced acquired natural immunity, and are likely to be most evident in relation to less resistant animals when infection pressure and nutritional stress are both high (Kidane et al., 2010), and therefore magnified in ewes feeding higher numbers of lambs. Therefore, twin-bearing ewes on a moderate plane of nutrition are probably the most suitable for studies on the effect of genotype on the PPR.

Benefits from selective breeding for nematode resistance may be realised in the maternal line through reduced egg output during the PPR, with positive epidemiological consequences on infection pressure in lambs, and without negative consequences for other aspects of performance. Measuring FECs at approximately 5–6 months of age for EBVs, as recommended by some breeding services, was observed to be an appropriate phenotypic parameter to select for resistance to gastrointestinal nematodes in maternal lines. Measuring FECs approximately 5 weeks after lambing appears to be an effective way of comparing egg output during the PPR in ewes.

The developmental success and survival of the free-living stages of *T. circumcincta* and *H. contortus* are predicted to improve in many regions as a result of climate change, increasing infection pressure. Predicted pasture infectivity from resistant and susceptible ewes under historic and a future climate change scenario in a temperate region in Europe demonstrated the potential of selection for resistance to gastrointestinal nematodes to mitigate the expected increase in challenge, and may offer a sustainable alternative or complement to chemical interventions.

## Appendix A Supplementary data

Supplementary data 1 Supplementary Fig. S1. Individual faecal egg count (FEC) trajectory for ewes with at least three FECs over the four sampling dates. The colours of the lines are varied to aid visualisation and do not represent any quantitative data elements. epg, eggs per gram of faeces. Supplementary Fig. S2. The frequency distribution of ewe estimated breeding values (EBVs) on Farm 1 and Farm 2. Supplementary Fig. S3. The simulated daily number of third-stage infective larvae (L3) derived from eggs excreted by ewes that are ingested by lambs over the course of a grazing season. The L3 are derived from eggs excreted by ewes assessed as having a high estimated breeding value (EBV) (nematode-susceptible; solid line) or low EBV (nematode-resistant; broken line). Supplementary Fig. S4. The simulated cumulative number of third-stage infective larvae (L3) derived from eggs excreted by ewes that are ingested by lambs over the course of a grazing season. The L3 are derived from eggs excreted by ewes assessed as having a high estimated breeding value (EBV) (nematode-susceptible; solid line) or low EBV (nematode-resistant; broken line).
