# Supplementary figures and images for: Reduced egg shedding in nematode-resistant ewes and projected epidemiological benefits under climate change
Source: Int J Parasitol. 2019 Nov;49(12):901–10. doi: 10.1016/j.ijpara.2019.06.008 (PMC6866873; doi:10.1016/j.ijpara.2019.06.008)

**Supplementary Fig. S1.**


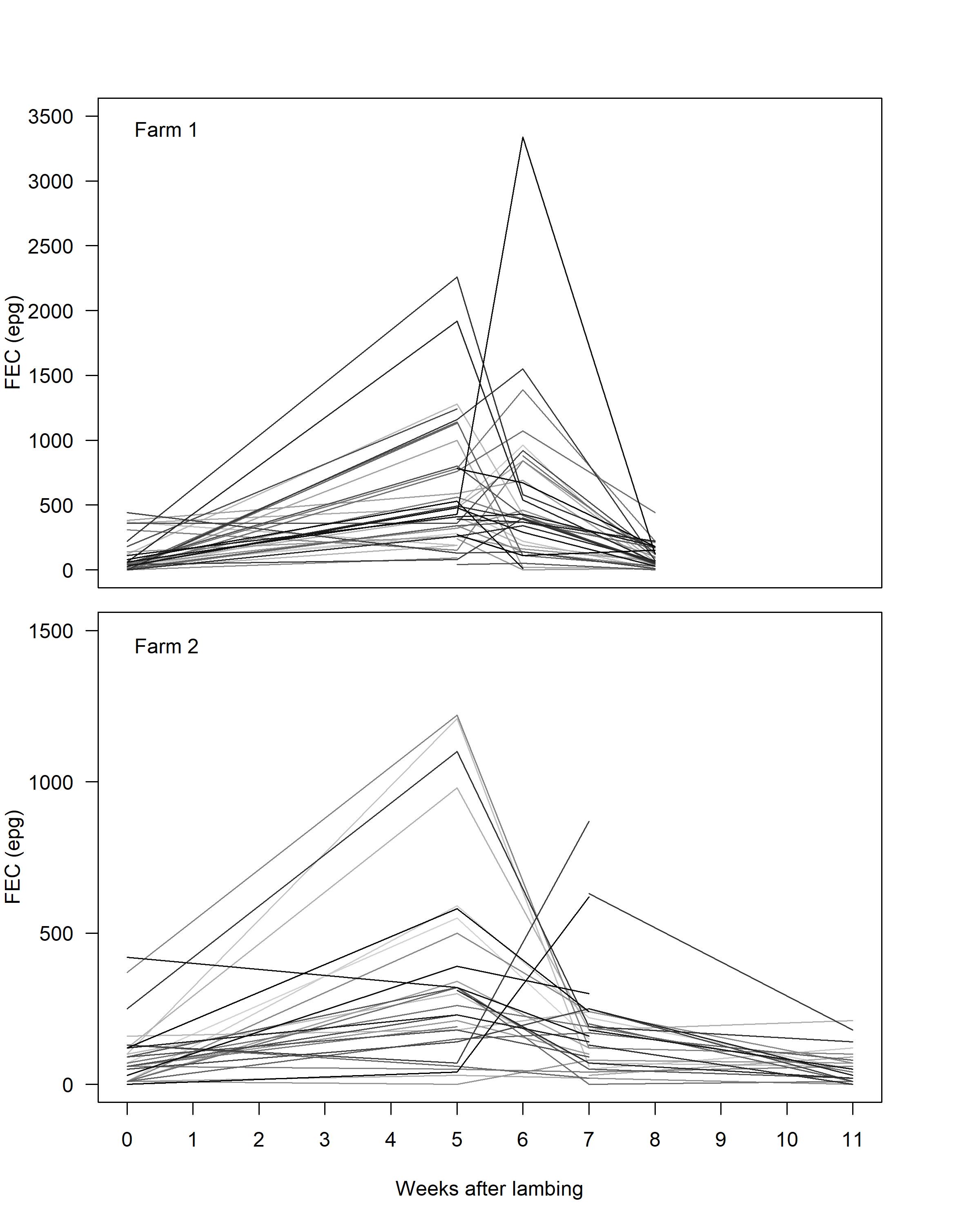


**Supplementary Fig. S2**.

*
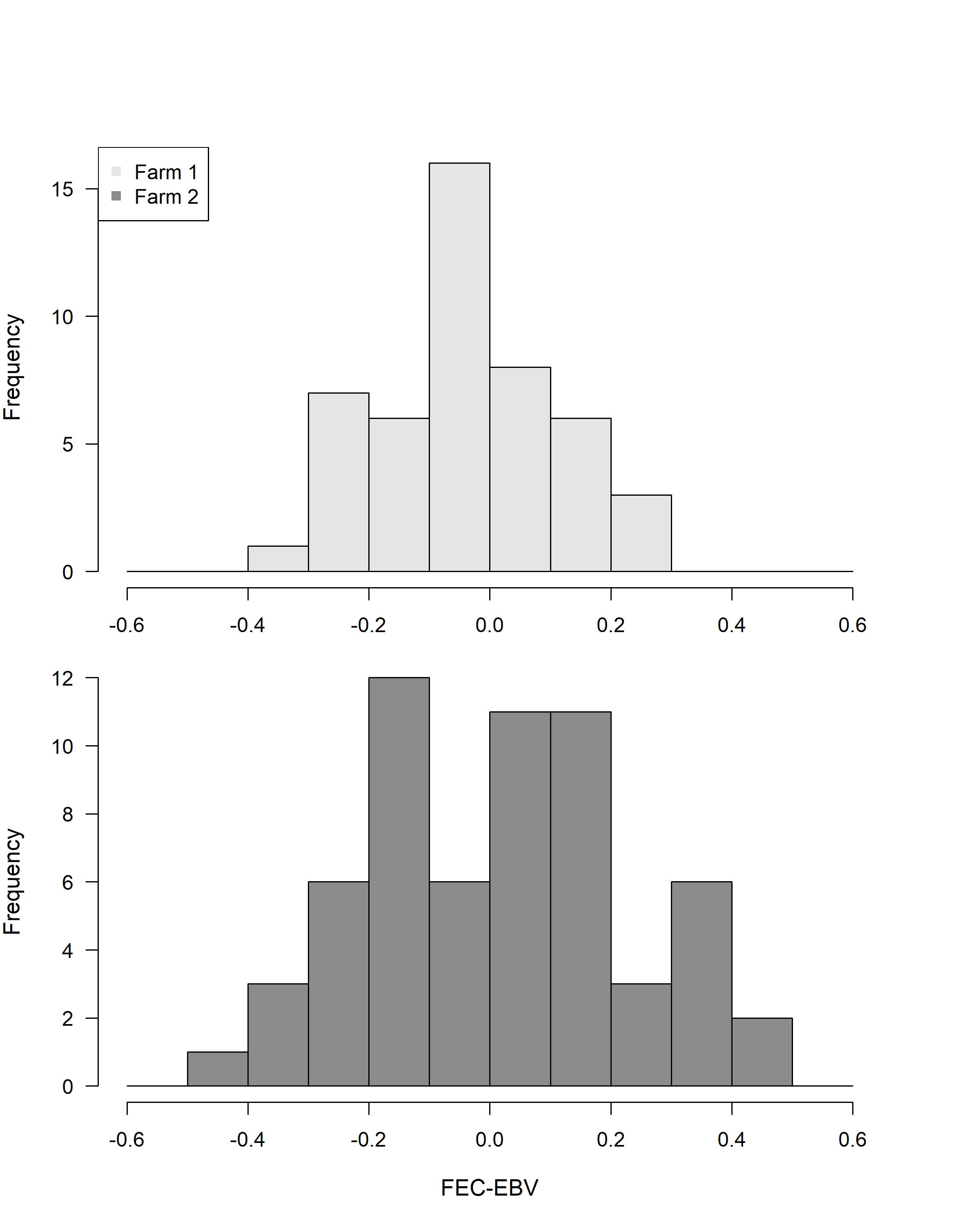
*

**Supplementary Fig. S3.**
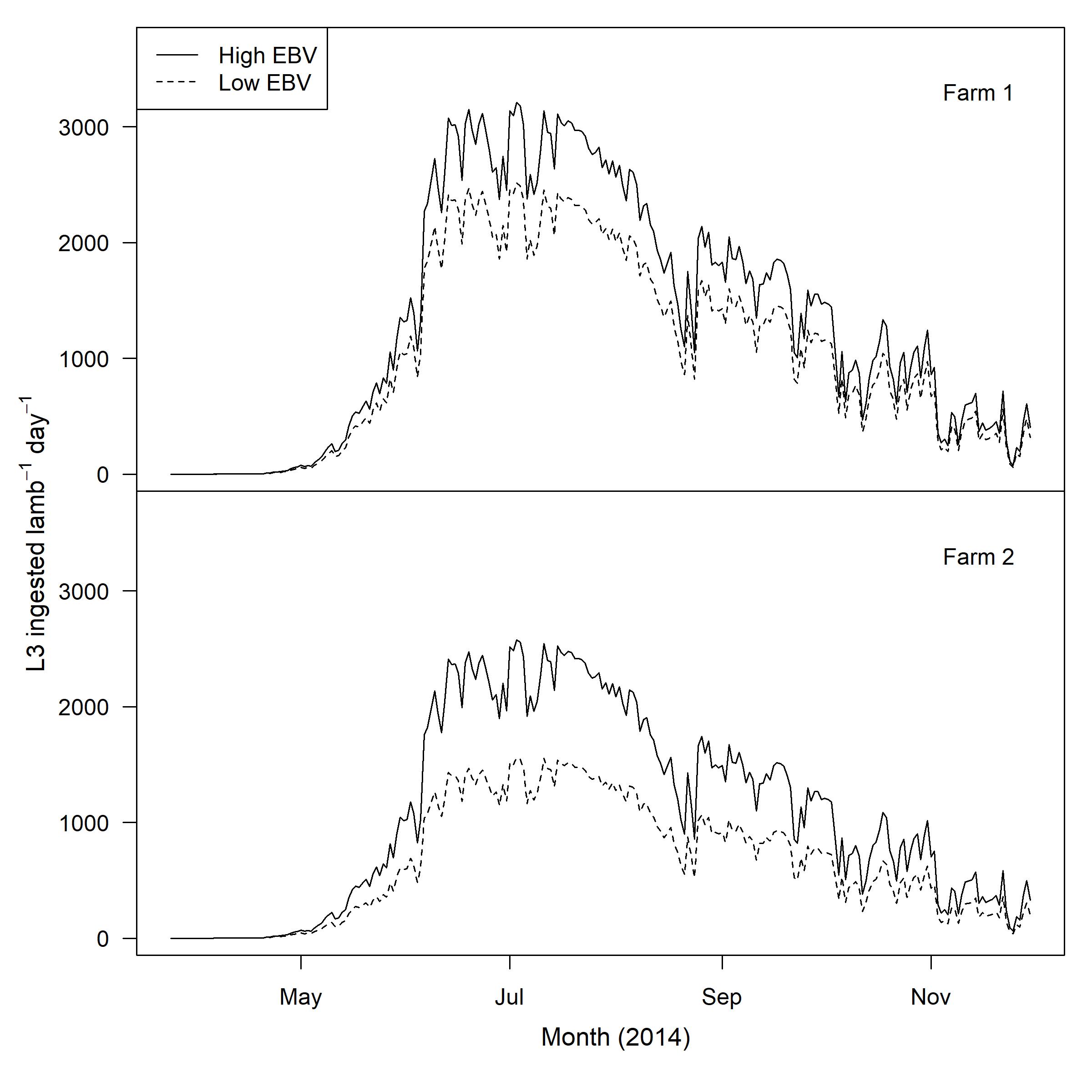


**Supplementary Fig. S4.**


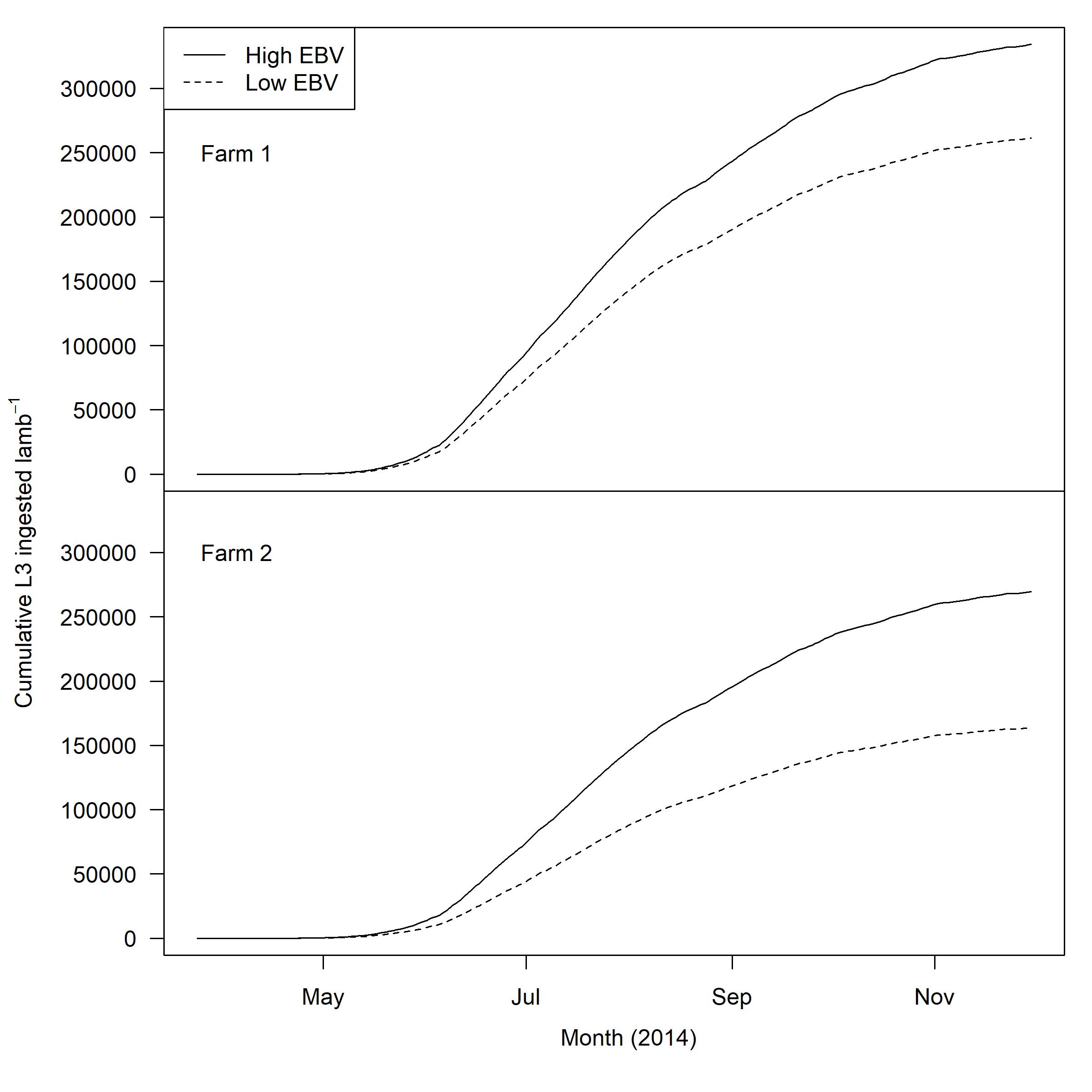

Supplement: Supplementary data 1 — Supplementary Fig. S1. Individual faecal egg count (FEC) trajectory for ewes with at least three FECs over the four sampling dates. The colours of the lines are varied to aid visualisation and do not represent any quantitative data elements. epg, eggs per gram of faeces. Supplementary Fig. S2. The frequency distribution of ewe estimated breeding values (EBVs) on Farm 1 and Farm 2. Supplementary Fig. S3. The simulated daily number of third-stage infective larvae (L3) derived from eggs excreted by ewes that are ingested by lambs over the course of a grazing season. The L3 are derived from eggs excreted by ewes assessed as having a high estimated breeding value (EBV) (nematode-susceptible; solid line) or low EBV (nematode-resistant; broken line). Supplementary Fig. S4. The simulated cumulative number of third-stage infective larvae (L3) derived from eggs excreted by ewes that are ingested by lambs over the course of a grazing season. The L3 are derived from eggs excreted by ewes assessed as having a high estimated breeding value (EBV) (nematode-susceptible; solid line) or low EBV (nematode-resistant; broken line). [file mmc1.docx]
