# Supplementary material for: Reduced egg shedding in nematode-resistant ewes and projected epidemiological benefits under climate change
Source: Int J Parasitol. 2019 Nov;49(12):901–10. doi: 10.1016/j.ijpara.2019.06.008 (PMC6866873; doi:10.1016/j.ijpara.2019.06.008)
Supplement: Supplementary data 2 [file mmc2.docx]

**Supplementary Table S1.** Farm 1 ewe estimated breeding values (EBV) and faecal egg counts (FEC) at intervals post-lambing.

|  | | **FEC** | | | | | | | | | |  |
| --- | --- | --- | --- | --- | --- | --- | --- | --- | --- | --- | --- | --- |
| **EBV** | | **Week 0** | | **Week 5** | | **Week 6** | | **Week 8** | | **Mean** | |  |
| -0.36 | | 40 | | 260 | |  | | 70 | | 123.3333333 | |  |
| -0.28 | | 30 | | 280 | |  | | 70 | | 126.6667 | |  |
| -0.27 | | 0 | | 490 | | 960 | | 0 | | 362.5 | |  |
| -0.25 | | 40 | | 480 | | 220 | | 10 | | 187.5 | |  |
| -0.24 | | 370 | | 190 | |  | | 10 | | 190 | |  |
| -0.22 | | 120 | | 1280 | | 430 | | 30 | | 465 | |  |
| -0.21 | | 10 | | 190 | |  | | 30 | | 76.66667 | |  |
| -0.2 | |  | | 480 | |  | | 70 | | 275 | |  |
| -0.17 | | 40 | | 410 | | 190 | | 70 | | 177.5 | |  |
| -0.17 | | 360 | | 480 | | 840 | | 60 | | 435 | |  |
| -0.13 | | 10 | | 1000 | | 20 | | 10 | | 260 | |  |
| -0.11 | | 380 | | 590 | | 690 | | 30 | | 422.5 | |  |
| -0.1 | |  | | 240 | | 0 | | 10 | | 83.33333 | |  |
| -0.1 | |  | | 880 | | 1870 | |  | | 1375 | |  |
| -0.09 | | 140 | | 260 | | 160 | | 70 | | 157.5 | |  |
| -0.09 | | 40 | | 320 | | 460 | | 100 | | 230 | |  |
| -0.07 | | 0 | | 90 | |  | | 10 | | 33.33333 | |  |
| -0.07 | | 0 | | 340 | | 140 | | 30 | | 127.5 | |  |
| -0.06 | | 310 | | 150 | | 840 | | 120 | | 355 | |  |
| -0.05 | | 40 | | 560 | | 390 | | 210 | | 300 | |  |
| -0.03 | | 40 | | 780 | | 1390 | | 220 | | 607.5 | |  |
| -0.02 | | 60 | |  | |  | | 60 | | 60 | |  |
| -0.02 | | 0 | | 760 | | 1070 | | 440 | | 567.5 | |  |
| -0.02 | | 80 | |  | | 880 | | 90 | | 350 | |  |
| -0.02 | | 0 | | 1130 | |  | | 80 | | 403.3333 | |  |
| -0.02 | | 20 | | 1140 | | 110 | | 30 | | 325 | |  |
| -0.02 | | 10 | | 340 | | 370 | | 170 | | 222.5 | |  |
| -0.02 | |  | | 40 | | 50 | | 0 | | 30 | |  |
| -0.02 | | 50 | | 800 | | 420 | | 40 | | 327.5 | |  |
| 0 | | 180 | | 1240 | |  | | 50 | | 490 | |  |
| 0.03 | | 360 | | 360 | | 920 | | 130 | | 442.5 | |  |
| 0.03 | |  | | 200 | |  | | 50 | | 125 | |  |
| 0.03 | | 440 | | 130 | | 140 | | 10 | | 180 | |  |
| 0.03 | | 40 | | 80 | | 400 | | 60 | | 145 | |  |
| 0.04 | |  | | 530 | |  | | 160 | | 345 | |  |
| 0.04 | | 0 | | 1160 | | 1550 | | 70 | | 695 | |  |
| 0.04 | | 220 | | 2260 | | 580 | | 170 | | 807.5 | |  |
| 0.06 | | 30 | | 490 | | 390 | | 50 | | 240 | |  |
| 0.14 | | 0 | | 260 | | 340 | | 70 | | 167.5 | |  |
| 0.14 | | 60 | | 1920 | | 540 | | 60 | | 645 | |  |
| 0.14 | |  | | 390 | | 370 | | 220 | | 326.6667 | |  |
| 0.15 | | 110 | | 410 | | 430 | | 180 | | 282.5 | |  |
| 0.16 | | 30 | | 480 | | 290 | | 30 | | 207.5 | |  |
| 0.18 | | 60 | | 430 | | 3340 | | 120 | | 987.5 | |  |
| 0.25 | | 80 | | 530 | | 10 | |  | | 206.6667 | |  |
| 0.26 | |  | | 780 | | 670 | | 180 | | 543.3333 | |  |
| 0.28 | |  | | 270 | | 110 | | 150 | | 176.6667 | |  |
| **Median** | | **40** | | **480** | | **410** | | **70** | |  | |  |
|  |  | |  | |  | |  | |  | |  | |

**Supplementary Table S2.** Farm 2 ewe estimated breeding values (EBV) and faecal egg counts (FEC) at intervals post-lambing.

|  | **FEC** | | | | |
| --- | --- | --- | --- | --- | --- |
| **EBV** | **Week 0** | **Week 5** | **Week 7** | **Week 11** | **Mean** |
| 0.2 | 30 |  | 10 |  | 20 |
| 0.09 | 70 | 550 | 150 |  | 256.6667 |
| 0.14 | 10 | 590 | 220 | 50 | 217.5 |
| -0.19 | 0 |  |  |  | 0 |
| -0.18 | 40 |  |  |  | 40 |
| -0.23 | 120 |  | 10 |  | 65 |
| -0.09 | 250 |  | 50 | 120 | 140 |
| -0.18 | 100 | 1210 | 60 |  | 456.6667 |
| 0.08 | 60 |  |  |  | 60 |
| 0.17 | 430 |  |  |  | 430 |
| 0.13 | 100 | 300 | 70 | 60 | 132.5 |
| 0.14 | 10 |  |  |  | 10 |
| -0.28 | 160 | 180 | 240 |  | 193.3333 |
| 0.08 | 120 | 980 | 180 | 210 | 372.5 |
| 0.2 | 10 | 30 | 20 |  | 20 |
| -0.24 | 90 |  | 30 | 90 | 70 |
| 0.11 | 10 | 340 | 120 |  | 156.6667 |
| -0.08 | 60 | 210 | 100 |  | 123.3333 |
| -0.11 | 10 | 0 | 80 | 70 | 40 |
| -0.14 | 20 |  | 20 | 0 | 13.33333 |
| -0.13 | 30 |  |  |  | 30 |
| 0.11 | 10 | 500 | 240 | 70 | 205 |
| 0.07 | 370 | 1220 | 120 | 100 | 452.5 |
| 0.12 | 30 |  | 200 |  | 115 |
| 0.14 | 60 | 50 | 40 | 60 | 52.5 |
| 0.07 | 60 | 190 |  | 70 | 106.6667 |
| 0.32 | 70 | 260 | 190 | 40 | 140 |
| 0.06 |  |  | 230 | 90 | 160 |
| 0.1 | 10 | 320 | 0 | 10 | 85 |
| 0.07 | 130 | 60 | 20 |  | 70 |
| 0.07 | 160 |  | 200 |  | 180 |
| -0.46 |  |  | 120 |  | 120 |
| -0.2 | 160 |  |  |  | 160 |
| -0.09 | 100 |  |  |  | 100 |
| -0.09 | 10 | 150 | 170 | 80 | 102.5 |
| 0.23 | 200 |  | 200 | 10 | 136.6667 |
| -0.22 | 20 |  |  |  | 20 |
| 0.32 |  |  | 530 |  | 530 |
| 0.24 |  | 1250 | 280 |  | 765 |
| -0.29 | 90 | 320 | 50 | 20 | 120 |
| -0.13 | 90 | 180 | 90 |  | 120 |
| -0.35 | 60 | 230 |  | 160 | 150 |
| -0.13 | 50 | 140 | 250 | 10 | 112.5 |
| 0.49 | 130 | 70 | 870 |  | 356.6667 |
| 0.17 | 600 |  | 630 | 180 | 470 |
| 0.27 | 250 | 1100 | 190 | 140 | 420 |
| 0.31 | 260 |  | 130 | 0 | 130 |
| -0.16 | 1130 |  | 290 |  | 710 |
| 0.32 |  | 1450 | 500 |  | 975 |
| -0.24 |  |  | 120 |  | 120 |
| -0.32 |  | 310 | 70 | 20 | 133.3333 |
| 0.34 | 160 |  | 250 | 30 | 146.6667 |
| -0.13 | 120 | 230 | 140 |  | 163.3333 |
| -0.1 |  |  | 170 | 80 | 125 |
| 0.3 | 330 |  |  |  | 330 |
| -0.31 |  | 240 |  |  | 240 |
| 0.41 | 430 |  | 180 | 50 | 220 |
| 0.05 | 420 | 320 | 160 |  | 300 |
| -0.05 | 70 |  | 110 |  | 90 |
| 0.31 |  |  | 330 | 70 | 200 |
| -0.08 | 30 | 390 | 300 |  | 240 |
| 0.09 | 120 | 580 | 240 |  | 313.3333 |
| -0.11 | 1180 |  |  |  | 1180 |
| -0.16 | 0 | 40 | 620 |  | 220 |
| 0.11 |  | 250 | 150 |  | 200 |
| **Median** | **90** | **280** | **160** | **70** |  |

**Supplementary Table S3.** Key production indicator data (ewe weight loss and lamb 8 week weights) for ewes on Farm 1 with known estimated breeding values (EBV) and faecal egg counts (FEC) recorded post-lambing. All ewes on this farm were carrying twins at scanning.

|  |  |  |  |  |  |  | **FEC** | | | | | |
| --- | --- | --- | --- | --- | --- | --- | --- | --- | --- | --- | --- | --- |
| **EBV** | **Number of lambs reared to 8 weeks** | **Mean 8 week lamb weight (kg)** | **Total 8 week lamb weight (kg)** | **Ewe liveweight before lambing (kg)** | **Ewe liveweight at weaning (kg)** | **Ewe liveweight loss (kg)** | **Week 0** | **Week 5** | **Week 7** | **Week 11** | **Mean** | **Max** |
| 0.2 | 2 | 22.5 | 45 | 59.4 | 38.6 | -20.8 | 30 |  | 10 |  | 20 | 30 |
| 0.09 | 2 | 24 | 48 | 62.6 | 52.8 | -9.8 | 70 | 550 | 150 |  | 256.6667 | 550 |
| 0.14 | 2 | 20.2 | 40.4 | 56.6 | 61.2 | 4.6 | 10 | 590 | 220 | 50 | 217.5 | 590 |
| -0.23 | 2 | 25 | 50 | 68.2 | 57.6 | -10.6 | 120 |  | 10 |  | 65 | 120 |
| -0.09 | 2 | 22.2 | 44.4 | 64.4 | 56.8 | -7.6 | 250 |  | 50 | 120 | 140 | 250 |
| -0.18 | 2 | 19.1 | 38.2 | 57.2 | 55.2 | -2 | 100 | 1210 | 60 |  | 456.6667 | 1210 |
| 0.13 | 2 | 25.9 | 51.8 | 65.8 | 54.4 | -11.4 | 100 | 300 | 70 | 60 | 132.5 | 300 |
| -0.28 | 1 | 31.6 | 31.6 | 59.4 | 54.4 | -5 | 160 | 180 | 240 |  | 193.3333 | 240 |
| 0.08 | 2 | 22.9 | 45.8 | 57.2 | 52 | -5.2 | 120 | 980 | 180 | 210 | 372.5 | 980 |
| 0.2 | 1 | 22.8 | 22.8 | 57.2 | 56.6 | -0.6 | 10 | 30 | 20 |  | 20 | 30 |
| -0.24 | 2 | 18.4 | 36.8 | 56 | 46.4 | -9.6 | 90 |  | 30 | 90 | 70 | 90 |
| 0.11 | 2 | 22.4 | 44.8 | 58.2 | 45.8 | -12.4 | 10 | 340 | 120 |  | 156.6667 | 340 |
| -0.08 | 2 | 24.1 | 48.2 | 61 | 53 | -8 | 60 | 210 | 100 |  | 123.3333 | 210 |
| -0.11 | 2 | 16.6 | 33.2 | 53.6 | 46 | -7.6 | 10 | 0 | 80 | 70 | 40 | 80 |
| -0.14 | 2 | 21.5 | 43 | 54.4 | 43.6 | -10.8 | 20 |  | 20 | 0 | 13.33333 | 20 |
| 0.11 | 3 | 14.13 | 42.4 | 58 | 46.4 | -11.6 | 10 | 500 | 240 | 70 | 205 | 500 |
| 0.12 | 2 | 17.4 | 34.8 | 56.2 | 50.4 | -5.8 | 30 |  | 200 |  | 115 | 200 |
| 0.14 | 2 | 19.3 | 38.6 | 59.2 | 51.4 | -7.8 | 60 | 50 | 40 | 60 | 52.5 | 60 |
| 0.07 | 2 | 21.9 | 43.8 | 57.8 | 54 | -3.8 | 60 | 190 |  | 70 | 106.6667 | 190 |
| 0.32 | 2 | 20.8 | 41.6 | 66 | 63.8 | -2.2 | 70 | 260 | 190 | 40 | 140 | 260 |
| 0.06 | 1 | 24.2 | 24.2 | 51.4 | 44.6 | -6.8 |  |  | 230 | 90 | 160 | 230 |
| 0.1 | 2 | 17.8 | 35.6 | 56 | 50.4 | -5.6 | 10 | 320 | 0 | 10 | 85 | 320 |
| 0.07 | 2 | 20.6 | 41.2 | 55.8 | 51 | -4.8 | 130 | 60 | 20 |  | 70 | 130 |
| 0.07 | 2 | 20.2 | 40.4 | 57.4 | 50.4 | -7 | 160 |  | 200 |  | 180 | 200 |
| -0.46 | 1 | 24.8 | 24.8 | 61.6 | 58.6 | -3 |  |  | 120 |  | 120 | 120 |
| -0.09 | 2 | 21 | 42 | 53 | 40.4 | -12.6 | 10 | 150 | 170 | 80 | 102.5 | 170 |
| 0.23 | 2 | 19.1 | 38.2 | 54.6 | 49.2 | -5.4 | 200 |  | 200 | 10 | 136.6667 | 200 |
| 0.32 | 2 | 22 | 44 | 61.2 | 52.4 | -8.8 |  |  | 530 |  | 530 | 530 |
| 0.24 | 2 | 11.6 | 23.2 | 49.2 | 50 | 0.8 |  | 1250 | 280 |  | 765 | 1250 |
| -0.29 | 1 | 25 | 25 | 49.4 | 48 | -1.4 | 90 | 320 | 50 | 20 | 120 | 320 |
| -0.35 | 2 | 20.5 | 41 | 54.6 | 49.6 | -5 | 60 | 230 |  | 160 | 150 | 230 |
| -0.13 | 2 | 17.8 | 35.6 | 54 | 50 | -4 | 50 | 140 | 250 | 10 | 112.5 | 250 |
| 0.49 | 2 | 16.4 | 32.8 | 63.8 | 69.2 | 5.4 | 130 | 70 | 870 |  | 356.6667 | 870 |
| 0.17 | 2 | 19.6 | 39.2 | 58 | 53.6 | -4.4 | 600 |  | 630 | 180 | 470 | 630 |
| 0.27 | 2 | 21.1 | 42.2 | 57 | 47.8 | -9.2 | 250 | 1100 | 190 | 140 | 420 | 1100 |
| 0.31 | 2 | 18.1 | 36.2 | 52.4 | 39.6 | -12.8 | 260 |  | 130 | 0 | 130 | 260 |
| -0.16 | 1 | 26 | 26 | 52.8 | 43.6 | -9.2 | 1130 |  | 290 |  | 710 | 1130 |
| 0.32 | 2 | 19.6 | 39.2 | 55.2 | 57.2 | 2 |  | 1450 | 500 |  | 975 | 1450 |
| -0.24 | 1 | 21.2 | 21.2 | 54.6 | 50.6 | -4 |  |  | 120 |  | 120 | 120 |
| -0.32 | 1 | 18.2 | 18.2 | 46.6 | 49.8 | 3.2 |  | 310 | 70 | 20 | 133.3333 | 310 |
| 0.34 | 2 | 17.6 | 35.2 | 54.4 | 48.6 | -5.8 | 160 |  | 250 | 30 | 146.6667 | 250 |
| -0.13 | 1 | 24.4 | 24.4 | 59 | 51.4 | -7.6 | 120 | 230 | 140 |  | 163.3333 | 230 |
| -0.1 | 1 | 24.4 | 24.4 | 56.4 | 41 | -15.4 |  |  | 170 | 80 | 125 | 170 |
| 0.41 | 1 | 26.6 | 26.6 | 55.6 | 45.2 | -10.4 | 430 |  | 180 | 50 | 220 | 430 |
| 0.05 | 1 | 19.4 | 19.4 | 57.8 | 52.6 | -5.2 | 420 | 320 | 160 |  | 300 | 420 |
| -0.05 | 2 | 20.4 | 40.8 | 64 | 54.8 | -9.2 | 70 |  | 110 |  | 90 | 110 |
| 0.31 | 2 | 20.7 | 41.4 | 51.8 | 45.8 | -6 |  |  | 330 | 70 | 200 | 330 |
| -0.08 | 1 | 23.2 | 23.2 | 51.4 | 55.8 | 4.4 | 30 | 390 | 300 |  | 240 | 390 |
| 0.09 | 1 | 23.4 | 23.4 | 61 | 50.6 | -10.4 | 120 | 580 | 240 |  | 313.3333 | 580 |
| -0.11 | 1 | 25 | 25 | 51.4 | 47.2 | -4.2 | 1180 |  |  |  | 1180 | 1180 |
| -0.16 | 2 | 20.4 | 40.8 | 50.6 | 45.4 | -5.2 | 0 | 40 | 620 |  | 220 | 620 |
| 0.11 | 1 | 22.6 | 22.6 | 54.4 | 43.8 | -10.6 |  | 250 | 150 |  | 200 | 250 |

**Supplementary Table S4.** Simulated cumulative exposure of lambs to *Teladorsagia circumcincta* third-stage infective larvae (L3) arising from eggs deposited by ewes during the peri-parturient rise. Exposure was based on daily estimates of L3 on herbage ha^-1^ (assuming a stocking rate of five ewes per hectare), a standing biomass of 2000 kg of dry matter (DM) ha^-1^ and daily herbage dry matter intake of growing lambs (Hynes, 2013). Scenario 1 simulated lamb exposure up to weaning only. Scenario 2 simulated set-stocking where lambs were grazed continuously on the lambing paddocks over an entire grazing season, and ewes were removed at weaning. High and low estimated breeding value (EBV) groups are defined in section 1 and 2.1 of the main text.

|  |  | **Exposure pre-weaning** | **Exposure throughout grazing season** |
| --- | --- | --- | --- |
| **Farm 1** | **High EBV** | 29,785 | 334,215 |
|  | **Low EBV** | 23,281 | 261,437 |
|  | **Difference in L3 ingested** | -6,504 | -72,778 |
|  | **% reduction** | -22 | -22 |
| **Farm 2** | **High EBV** | 23,537 | 269,674 |
|  | **Low EBV** | 13,998 | 163,748 |
|  | **Difference in L3 ingested** | -9,539 | -105,927 |
|  | **% reduction** | -41 | -39 |

Reference

Hynes, F., 2013. Sheep technical note April 2013: Grass and fodder shortage. Available at: http://www.teagasc.ie/publications/2013/1907/GrassScarcityLactatingEwe.pdf
